# Optimization Design of Quenching and Tempering Parameters for Crankshaft Based on Response Surface Methodology

**DOI:** 10.3390/ma17153643

**Published:** 2024-07-24

**Authors:** Yongkang Wang, Jie Tang, Jianzhi Chen, Zhibin Nie, De Zhao

**Affiliations:** 1 School of Mechanical Engineering, Jiangsu University of Science and Technology, Zhenjiang 212003, China; jskjwyk77@163.com (Y.W.);; 2 Henan Diesel Engine Heavy Industry Co., Ltd., Luoyang 471039, China

**Keywords:** finite element simulation, crankshaft, quenching and tempering, response surface method

## Abstract

Existing optimization research on the crankshaft heat treatment process is mostly based on one-sided considerations, and less consideration is given to the matching of multiple process parameters, leading to irrational designs of heat treatment. To address this problem, this work investigates the influence mechanisms of cooling speed, tempering temperature, and holding time on the performance evaluation indexes of the straightness, residual stress, and martensite content of a crankshaft based on the response surface method. The results showed that the order of influence of these three different process parameters on the performance evaluation index was cooling speed > holding time > tempering temperature, and the order of influence on the performance evaluation indexes under multifactorial process parameters was cooling speed–holding time > cooling speed–tempering temperature > holding time–tempering temperature. The optimal process parameters were a cooling speed of 1.4 times the cooling oil, a tempering temperature of 555 °C, and a holding time of 6 h, with the straightness of the crankshaft reduced by 9.9%, the surface stress increased by 6.7%, and the martensitic content increased by 7.2% after the process optimization. This work can provide new clues for optimizing the heat treatment process parameters of crankshafts.

## 1. Introduction

A crankshaft is one of the most important components used in marine diesel engines, and its performance directly influences the life-span of the diesel engine [1,2,3]. The quenching and tempering process, as the basic heat treatment for the crankshaft, is often used to adjust its microstructure and mechanical properties [4,5,6,7]. In the heat treatment of quenching and tempering, different parameters such as cooling speed, tempering temperature, and holding time will affect the heat treatment evaluation indexes of the straightness, residual stress, and phase composition of the crankshaft, thereby influencing its mechanical properties. So, studying the influences of these heat treatment parameters on the evaluation indexes is of great significance for optimizing the mechanical performance of the crankshaft.

The mechanical properties of the crankshaft after the quenching and tempering heat treatment are affected by process parameters such as the cooling speed, tempering temperature, and holding time. Among them, in terms of the impact of the quenching process on steel properties, Dai et al. [8,9,10] studied the impact of the quenching process on the mechanical properties of steel, and found that the phase transformation during the quenching stage of the crankshaft and the extended heating time could improve the uniformity of the crankshaft surface temperature and the thickness of the hardened layer. SG and Yang et al. [11,12] investigated the effect of the tempering temperature on the microstructure distribution and tensile properties of 17Cr2Ni2MoVNb steel, and the results showed that the tensile properties of the specimens with tempering temperatures of 180 °C and 150 °C were the best, with tensile strengths of 1456 MPa and 2133 MPa, respectively. In addition to affecting the tensile properties of the steel, the tempering temperature also affects other mechanical properties of the steel. Ren et al. [13] studied the influence mechanism of the tempering temperature on the microstructure and mechanical properties of 35CrMo steel, and found that the strength of 35CrMo steel decreased and the toughness increased with increments in the tempering temperature in the temperature range of 580–680 °C. Ma et al. [14] studied the effect of the tempering temperature on the precipitation behaviors of heat-resistant steel, and found that the fracture exhibited an obvious brittle fracture when the tempering temperature was lower than or equal to 550 °C, with an obvious ductile fracture forming when the temperature was higher than 550 °C. Arabacı et al. [15] studied the impact of the oil quenching and tempering heat treatment on the wear properties of 25CrMo4 steel, and found the dry sliding wear losses decreased significantly in the oil-quenched and over-tempered samples compared to the raw material. Murdoch et al. [16] employed a simple Gaussian process regressor model to predict the steel hardness and toughness response of tempered martensitic steels and utilized the Shapley additive explanations to assess the importance of the input features of the tempering temperature, tempering time, and other 15 factors. The tempering temperature and carbon content were found to be the most important input features considering all factors. The quenching and tempering heat treatment process of the crankshaft is affected by a series of parameters such as cooling speed, quenching temperature, holding time, and tempering temperature [17]. Previous related research has usually only considered a single factor, such as the impact of cooling speed on the crankshaft quenching and tempering process or using simulation experiments to study the effect of one process parameter on the performance of the crankshaft, rarely analyzing the impact mechanisms of combined parameter combinations on the straightness, surface residual stress, hardness, and other performance evaluation indexes.

In this work, the effects of cooling speed, tempering temperature, and holding time on the deformation, residual stress, and microstructure of a crankshaft were analyzed based on a combination of simulations and experimental verification. The best quenching and tempering process parameter corresponding to superior heat treatment qualities of the crankshaft was acquired. The results and methods in this work can provide new clues for the optimization of the heat treatments of mechanical parts.

## 2. Description of Research Methods

### 2.1. Analysis of Crankshaft Quenching and Tempering Heat Treatment Process

**Figure 1:**
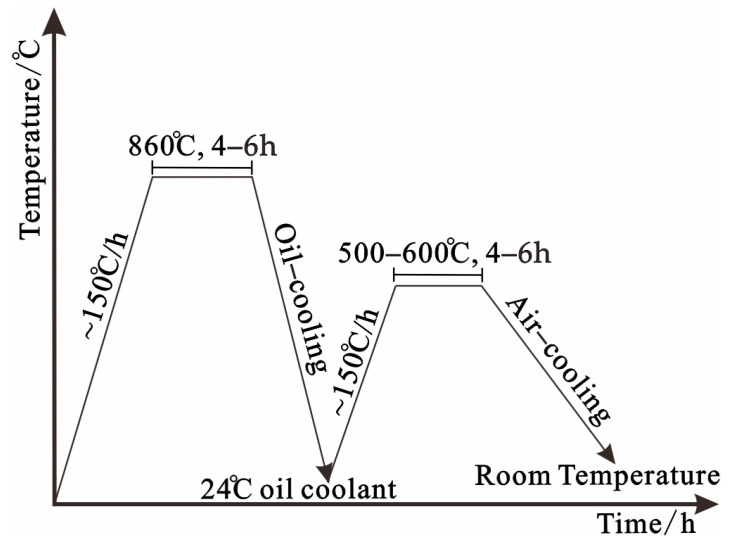
Quenching and tempering heat treatment route of the crankshaft.

At present, the quenching and tempering heat treatment process of crankshafts is commonly used in factories. The crankshaft is first heated to 860 °C and kept warm for 4–6 h, and then cooled in a coolant of oil for 10 min, with the temperature of the coolant being 24 °C. The cooling speed varies greatly depending on the material. After the quenching process, high-temperature tempering with a temperature between 500 and 600 °C and a holding time of 4–6 h is performed. The basic route for the quenching and tempering heat treatment process of crankshafts is shown in Figure 1.

### 2.2. Simulation Principle

The software COMSOL Multiphysics 6.1 (COMSOL Company, Stockholm, Sweden) was utilized in the simulation. COMSOL is based on the finite element method, which is able to simulate and analyze a variety of physical phenomena and engineering problems with multi-physics field-coupling capabilities, and can be integrated into different physical fields in a unified model, allowing users to solve interdisciplinary complex problems in the same simulation environment. In this simulation, the elastic–plastic properties of each metallographic phase were regarded as linear elasticity with linear hardening. The linear elastic properties were given by the Young’s modulus and Poisson’s ratio of the individual phases, and the plastic properties were given by the initial yield stress and the isotropic hardening modulus. The Austenite Decomposition [18] physics interface automatically averaged these properties into effective properties that defined the composite material. Like thermal analysis, the mechanical analysis involved material properties related to temperature and phase composition. In this simulation, it was assumed that the elastic properties of the phases were equal. The secant coefficient of thermal expansion was not averaged into the multiphase material properties, but was used to calculate the thermal strain tensor for each metallographic phase [19].

The thermal strain tensors were then averaged to obtain the thermal strain of the multiphase material. In order to complete the description of the phase properties, a volume reference temperature was defined for each metallographic phase. The choice of volumetric reference temperature is somewhat arbitrary. In this simulation, we did not explicitly consider the heating phase (austenitization), so the volume reference temperature was set to the austenitization temperature (900 °C), meaning that the crankshaft was strain-free at this temperature. To account for the strains resulting from the thermal expansion and austenitization of the base phase composition, an initial strain was applied in the simulation. To account for the strains resulting from the thermal expansion and austenitization of the base phase composition, we applied an initial strain, which is given by:

(1) $$\varepsilon_{0}=5\times 10^{-3}\left(\begin{array}{ccc} 1 & 0 & 0\\ 0 & 1 & 0\\ 0 & 0 & 1 \end{array}\right)$$

The determination of the cooling speed is related to the cooling function, which is re placed by the heat transfer coefficient of *h* related to the temperature. According to this coefficient, we can obtain:

*q* = *h*(*T*)·(*T*_0_ − *T*) (2)

Among them, *T*_0_ = 24 °C, the heat flux is *h*(W/(m^2^·K)), and the thermal conduction function table was obtained. Solid mechanics were added to the software of COMSOL 6.1 to analyze residual stress and strain, and the austenite decomposition physical field was added to analyze the changes in the various microstructures in the crankshaft after the quenching and tempering treatment. Material properties were multi-phase material properties, and the properties were composed of austenite, ferrite, pearlite, bainite, and martensite. Here, we define the mechanical properties of the materials according to the mixing rule and define the reference material by the means of the material properties. This simulation experiment simulated the quenching and high-temperature tempering heat treatment of a crankshaft made of 34CrNiMoA alloy steel.

During the cooling process, the austenite can decompose into a combination of ferrite, pearlite, bainite, and martensite. The phase transition to martensite is displacement and can be described by the Koistinen–Marburger model, where the amount of martensite formed at the expense of austenite at the surface depends on the ratio column of available austenite and the degree of subcooling below the martensite onset temperature *M*_s_ [20]. The model is given by the following equation:

*£*^d^ = −*£*^s^*ßT* (3)

where the rate of martensite formation is proportional to the temperature rate and the instantaneous ratio of austenite, with the scale factor being the Koistinen–Marburger model coefficient *ß*. The remaining diffusive phase transition was modeled using the Leblond–Devaux model [21]. The model is given by the following equation:

*£*^d^ = *K*(*T*)*£*^s^ − *L*(*T*)*£*^s^ (4)

The crankshaft quenching and high-temperature tempering heat treatment was analyzed by the above simulation principle.

**Figure 2:**
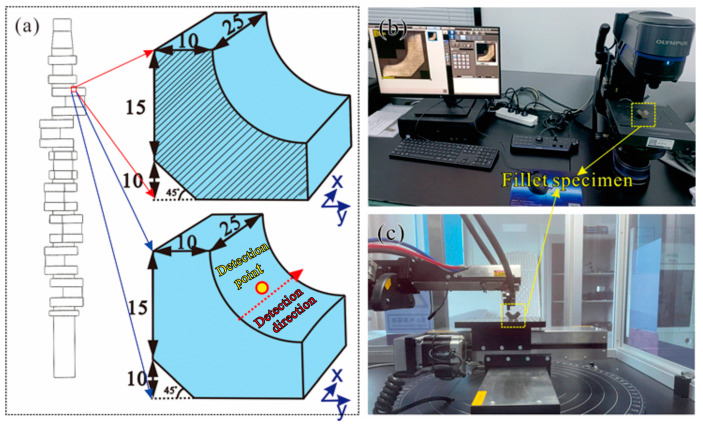
(**a**) Schematic diagrams of the specimens for metallographic characterization and residual stress detection, (**b**) experimental photo of the metallographic characterization, and (**c**) experimental photo of the residual stress detection.

In addition, in order to verify the accuracy of the simulation, a crankshaft under the factory process parameters of a quenching temperature of 860 °C, tempering temperature of 550 °C, and holding time of 5 h was prepared. The microstructure in the fillet area of the crankshaft was cut and tested, and the observation section of the fillet specimen is shown by the shaded area in Figure 2a. The observation section was ground and polished, and then etched by using the 4% nital. The microstructure was observed by an Olympus microscope (DSX1000), as shown in Figure 2b. The volume fraction of martensite in the microstructure of the fillet area was measured and compared with the simulated results. The X-ray diffractometer (I-XRD COMBO) shown in Figure 2c with Cr Kα radiation and a 0.5 mm collimator was used to acquire the residual stress (*σ*_res_) on the fillet surface, and the detection point is indicated by the yellow circle in Figure 2a. The residual stress on the surface of the fillet (along the dotted red arrow direction) was also compared with the simulated result to verify the accuracy of the simulation method. 

### 2.3. Establishment of Response Surface Method

Response surface analysis, the full name of the response surface design method, is a specific experimental design method that aims to explore the impacts of multiple control variables on response variables through a smaller number of experiments. The response surface method is based on the multiple regression analysis and fits experimental data to a mathematical model to predict the responses of the response variables to each control variable. Two common response surface method experimental design approaches are CCD (central composite design) and BBD (Box–Behnken). The BBD design is suitable for response surface optimization experiments with three or four factors. This design can quickly and effectively fit the multiple regression model of the response variable by setting the experimental points at different levels around the central point [22].

CCD is a common three-level full factorial design method in response surface methodology which is used to determine the linear, quadratic, and interactive effects of response variables on factors. CCD’s experimental protocols are suitable for mathematical models where the experiments are non-linear and include full factorial experimental designs, axial point experimental designs, and horizontal center point experimental designs. Through the CCD design, researchers can fully understand the impacts of multiple factors on response variables and determine the optimal experimental conditions [23]. Both experimental design methods have the advantages of a high accuracy and good predictability. These common response surface methodology experimental design methods can help researchers to systematically explore the impacts of multiple factors on response variables to optimize the performance processes. The response surface refers to the functional relationship between the response variable *η* and a set of input variables (*ζ*1, *ζ*2, *ζ*3, …, ζk): *η* = *f*(ζ1, ζ2, ζ3, …, ζk) [24,25].

When the quenching and tempering process was completed, the deformation of the crankshaft workpiece was large, and the surface hardness may not meet the standard. So, in order to reduce the deformation and improve the hardness of the workpiece, the cooling speed, the tempering temperature and the holding time were selected as the variables of the experiments, the straightness, the surface residual stress and the martensite content of the crankshaft after the heat treatment were selected as the optimization goals. Table 1 shows the factor levels. The CCD method of three process parameters (X1, X2, and X3) was further used for experimental planning, which was carried out based on a finite element analysis of the quenching and tempering heat treatment. Among them, the three different levels of “low”, “medium”, and “high” were replaced by “−1”, “0”, and “1”. The factor levels, shown in Table 1, indicate the levels of the actual variation intervals of the three process parameters of cooling rate, tempering temperature, and holding time corresponding to the experimental planning levels. Table 2 demonstrates the specific experimental scheme and results by varying the values of the process parameters to obtain the corresponding performance index simulation results.

## 3. Results and Analysis

### 3.1. Analysis of Simulation Results

**Figure 3:**
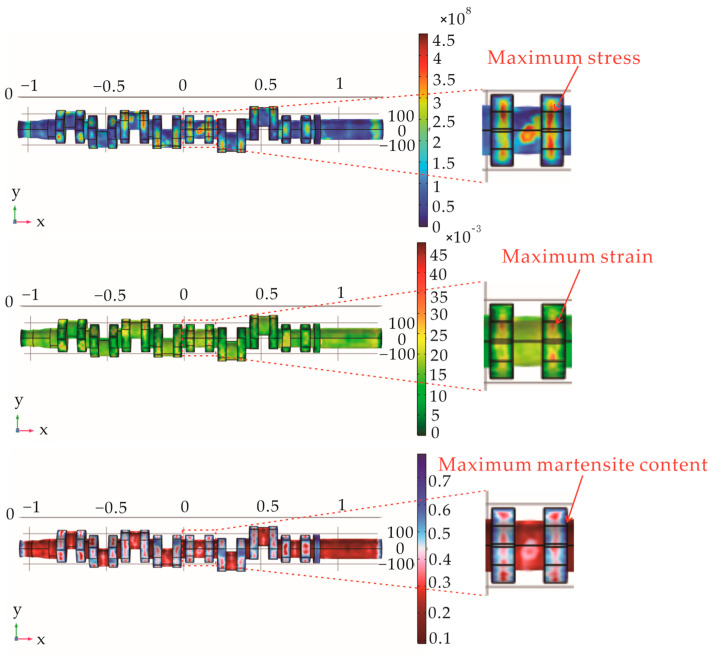
Simulated cloud maps of (**a**) residual stress, (**b**) residual strain, and (**c**) martensite content of the crankshaft after the quenching and tempering heat treatment.

Under the factory process parameters of a quenching temperature 860 °C, high-temperature tempering temperature of 550 °C, and holding time of 5 h, through the COMSOL software finite element simulation, the crankshaft quenching and tempering heat treatment results were obtained, as shown in Figure 3. The maximum value of the residual surface stress was at the outer surface of the crankshaft, with a maximum value of 462 MPa. For engines with a high speed and high load, a higher residual stress is beneficial for resisting fatigue fracture, improving fatigue strength, and prolonging fatigue life. The appropriate residual stress can help to resist the deformation due to thermal expansion and contraction, as well as cyclic loading in the process of operation, in order to enhance the stability of the structure. The residual stress distribution in the crankshaft and crank parts gradually decreased from the middle crank of the crankshaft to both sides. The overall distribution of residual stress was more uniform, which can make the crankshaft reduce the abnormal vibration in the working process, reduce the noise, and make it difficult to trigger the resonance phenomenon, so as to ensure the stable operation of the engine. The residual stress distribution of the crankshaft was basically consistent with that of the actual crankshaft after the tempering heat treatment, and also consistent with the residual stress distribution on the crankshaft surface in most crankshaft performance studies and analyses [26].

The analysis of the microstructure distribution results showed that the martensite distribution was higher at the crank after the crankshaft heat treatment, the maximum martensite content value of 0.7639 is observed on the surface of the crank and a lower content of martensite exists in the areas of rod journal. The minimum value appeared on the front and rear end shafts, and the minimum martensite content was 0.1. The martensite content on the outer surface of the crank was higher than the martensite content on the inner side, the fillet was between the crank and the connecting rod journal, and the values were relatively balanced. The martensite content has a significant effect on the mechanical properties of crankshafts, because the martensite phase is a harder and stronger phase, and the hardness and strength of crankshafts usually increase with an increase in the martensite volume fraction. The formation and distribution of martensite have an important effect on the fatigue life of crankshafts. Under the cyclic loading, the presence of a martensite phase may accelerate the formation and extension of fatigue cracks, especially in the region of a high stress concentration. Therefore, the optimization of the martensite volume fraction is also crucial for improvements in the fatigue lives of crankshafts, and in this study, we chose to use the martensite content of the crankshafts in the simulation results to represent the hardness condition of the crankshafts. In view of the experimental target, the fillet between the crank and the journal was selected as the martensite content selection point, and the required experimental data were recorded.

After analyzing the residual deformation, it was found that the strain in each part of the crankshaft was greater in the axial direction than in the other directions, the strain at the connection between the connecting rod journal and the crank was large, and the overall deformation of the crank was small.

**Figure 4:**
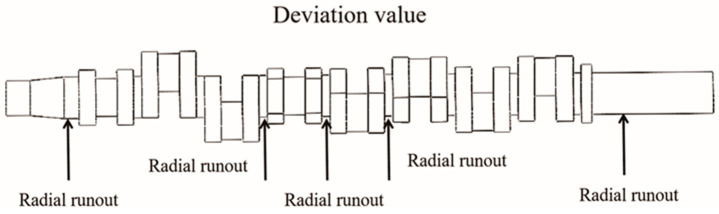
Crankshaft straightness deviation measurement chart.

**Figure 5:**
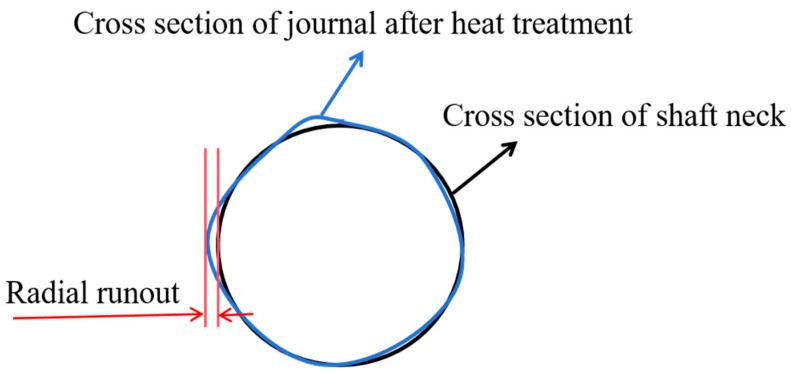
Schematic diagram of radial runout measurement.

Straightness refers to the straightness of an object’s surface, that is, the deviation between the object’s surface and an ideal straight line. Based on the simulation results, the deformation of the front shaft of the crankshaft, the three main journals, and the flange at the rear end of the crankshaft were selected to determine the axis deviation at the front and rear ends, and the radial runout value of the journal, the axis deviation difference, and the radial runout were obtained. The measured values are shown in Figure 4 and Figure 5. Figure 4 measures the radial runout at five positions, and Figure 5 demonstrates the radial runout as the deformation of the crankshaft end face before and after the heat treatment. The crankshaft straightness was determined based on the axis deviation difference [27]. The straightness under this process was 9.83 mm.

**Figure 6:**
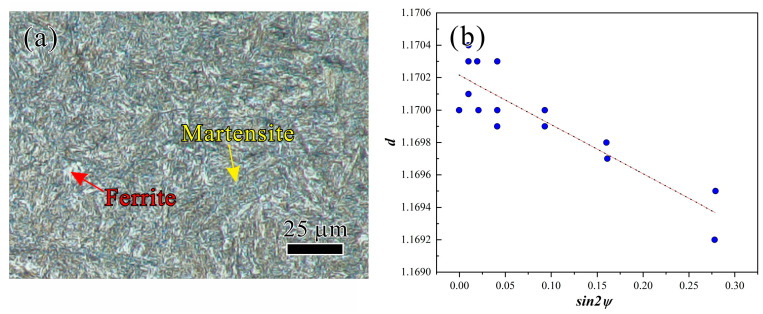
(**a**) Microstructure in the fillet area of the crankshaft and (**b**) relationship between *d* and sin^2^*ψ*.

In order to verify the accuracy of the simulation method, the microstructure in the fillet area of the crankshaft (Figure 2a) was observed, as shown in Figure 6a. The volume fraction of martensite in the microstructure of the fillet area was measured based on the point-counting method, and the measured volume fraction of martensite was ~0.73. The simulation result of 0.77 shown in Figure 3 exhibited a deviation of 5.48% when compared with the measured results. An X-ray diffractometer was utilized to measure the residual stress on the surface of the fillet, and the relationship between the *d* and sin^2^*ψ* was obtained as shown in Figure 6b, where *d* is the interplanar crystal spacing and *ψ* is the azimuth angle of the crystal plane. A residual stress of −499.26 MPa was obtained based on the data of Figure 6b and the method of sin^2^*ψ* reported in other work [28], and the simulated result of −462 MPa showed a deviation of 7.46% when compared with the detected result. By comparing the test results with the simulation results, we can draw the conclusion that our simulation method had a high accuracy.

### 3.2. Response Surface Method Analysis

Using the Design-Expert design factor prediction model, the targets were *D* (crankshaft straightness), *K* (surface stress), and *M* (martensite content), and the entire experimental results were regression analyzed by the least squares method [29]. Experimental designs usually use the size of the multiple fitting coefficient *R*^2^, the modified fitting coefficient *R*^2^_adj_, and the predicted fitting coefficient *R*^2^_pre_ to evaluate and verify the goodness of the regression model fit. Usually, the range of *R*^2^ and *R*^2^_adj_ changes between 0 and 1 [30,31]. When *R*^2^ and *R*^2^_adj_ are closer to 1, the smaller the difference between the predicted fitting coefficient *R*^2^_pre_ and the modified fitting coefficient *R*^2^_adj_, the better the fit between the regression function and the model. Through a data analysis of the software of Design-Expert 7.0, the accuracy of the polynomial function model under different process conditions was compared, as shown in Table 3, with the fitting accuracy of the regression models of *D* (crankshaft straightness), *K* (surface stress), and *M* (martensite content), respectively.

As shown in the above table, *D* (crankshaft straightness), *K* (surface stress), and *M* (martensite content) had the best fitting performance of the model under the conditions of the second-order equation. The fitting coefficient values of *R*^2^ and the modified fitting coefficient of *R*^2^_adj_ for *D* were 0.9072 and 0.8858, respectively, the fitting coefficient of *R*^2^ and the modified fitting coefficient of *R*^2^_adj_ for the surface stress *K* were 0.8704 and 0.840, and the fitting coefficient of *R*^2^ and the modified fitting coefficient of *R*^2^_adj_ for the martensite content *M* were 0.8758 and 0.8471, respectively. All three evaluation indexes were close to 1, indicating that this model had a high prediction accuracy and reliability. Comparing the data in the table, it was finally determined that the response prediction model of the crankshaft straightness, surface stress, and martensite content was a polynomial function under the condition of a second-order equation, as shown in Equations (5)–(7).

*D* = 9.83 − 2.56A − 0.265B − 1.11C − 0.0475AB − 0.15AC − 0.0575BC − 1.12A^2^+0.035B^2^ − 0.1775C^2^ (5)

*K* = 4.62 + 0.42A + 0.0363B + 0.091C − 0.0025AB + 0.0275AC + 0.025BC + 0.0275A^2^ − 0.21B^2^ − 0.06C^2^ (6)

*M* = 76.23 + 12.05A + 0.9275B + 2.73C − 0.13AB + 1.2AC + 0.12BC − 0.6138A^2^ − 5.12B^2^ − 3.16C^2^ (7)

The coefficient size before each term in the above formula indicates the degree of influence of each process influencing factor on the response surface model, and the positive and negative in the function indicate the direction. After obtaining the regression equation, the accuracy of the model needs to be judged and verified again to determine the degree of fit between the model and the equation. Table 4 shows the significance judgment of the equation.

**Figure 7:**
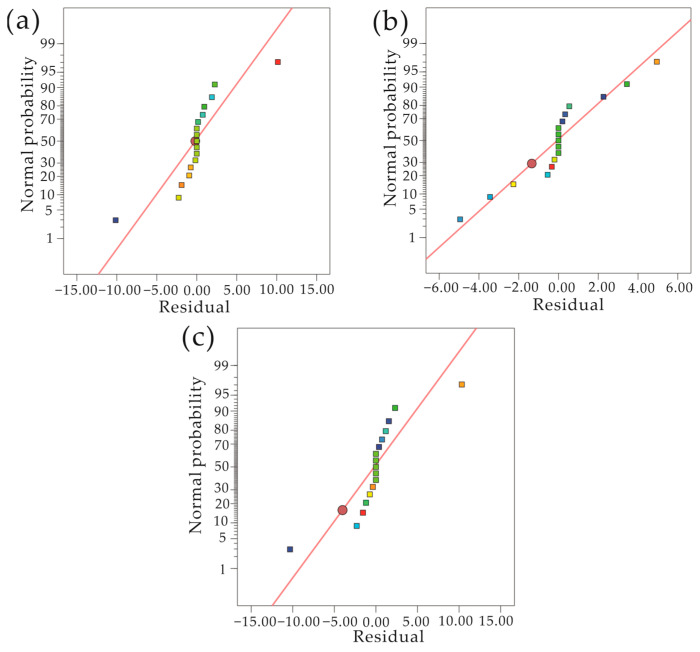
Residual normal distribution diagram. (**a**) Crankshaft straightness model residual normal distribution. (**b**) Surface stress model residual normal distribution. (**c**) Martensite content model residual normal distribution.

When the model probability is *p* > 0.1, the model effect can be considered to be insignificant. When the model probability is *p* < 0.01, the model effect can be considered to be significant. Figure 7 shows the normal distribution diagram of the residuals of the crankshaft straightness model, surface stress model, and martensite content model. From the figure, we can see that the residuals of these three models are basically on a straight line, indicating that the model conformed to the law of normal distribution. Combining all the above data and evaluation standards, we can see that, through the optimized design of process parameters for the quenching and tempering heat treatment of the crankshaft, the completed response surface model had a good fitting ability and high prediction accuracy.

The target parameter prediction model and the variance analysis diagram of each process parameter shown in Table 5, Table 6 and Table 7 can provide a clearer understanding of the impact of each parameter condition on the crankshaft straightness. *F* is the ratio of the experimental treatment effect and the error effect. If *F* < 1, it means that the data are mostly caused by individual errors and experimental errors, that is to say, there is not much difference between the different experimental treatments. If *F* = 1, it means that the difference in experimental treatments is not big enough. If *F* > 1, it indicates that the data are basically caused by the experimental processing, that is, there are obvious differences in the different experimental treatments [32,33]. As can be seen from Table 5, Table 6 and Table 7, the *F* values of each prediction model were 64.36, 156.16, and 326.26 respectively, so it can be understood that the model differences were obvious. *p* is the significance level, and the *p* value of each prediction model was below 0.0001, which can be considered as significant.

**Figure 8:**
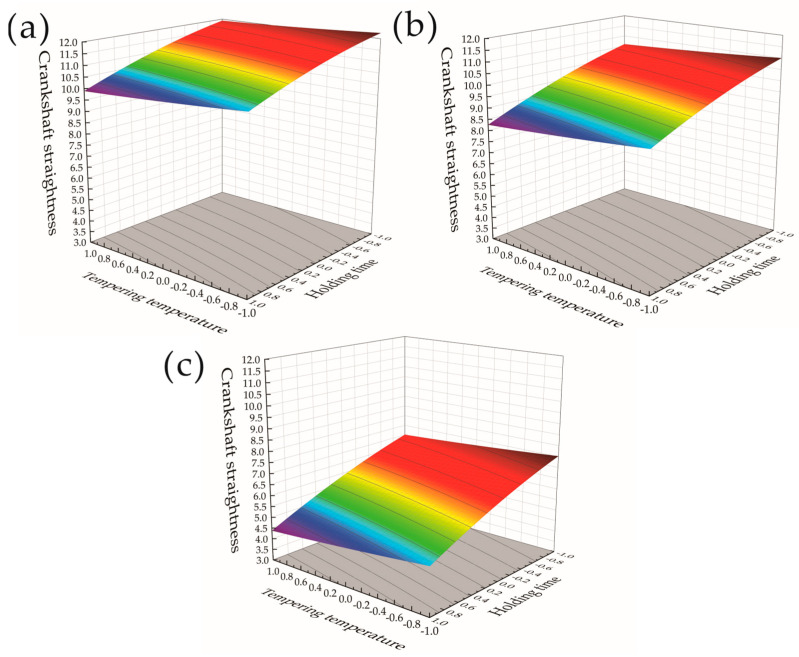
Response surface and contour plot of tempering temperature and holding time to crankshaft straightness. (**a**) Cooling oil with a cooling speed of 1 times. (**b**) Cooling oil with a cooling speed of 1.2 times. (**c**) Cooling oil with a cooling speed of 1.4 times.

The influence of changes in the crankshaft heat treatment process parameters on the crankshaft straightness was analyzed, and the response surfaces and contour plots of different heat treatment parameters on the crankshaft straightness were obtained to more intuitively see the effects of each factor on the response value at different experimental factor levels. The response surface contour color does not have much other meaning, and this is used to indicate the value of the high and low changes in the situation, with a more intuitive representation of the trend of numerical changes in the process. Figure 8a–c show the response surfaces of the effects of the tempering temperature and holding time on the crankshaft straightness when the cooling speed was 1, 1.2, and 1.4 times that of the cooling oil, respectively, and contour maps. It can be seen from Figure 8a,b that, when the cooling speed was high, the straightness of the crankshaft was smaller. Figure 8a–c show that along the tempering temperature and holding time −1 to 1 horizontal line, an increase in the tempering temperature and holding time will also reduce the straightness of the crankshaft. Among them, the tempering temperature had a smaller impact on the straightness of the crankshaft. As shown in Figure 8a, along the tempering temperature horizontal line, the crankshaft decreased slightly. It can be seen from Figure 8c that the overall deformation was small and the crankshaft straightness became smaller when the crankshaft cooling speed increased. Therefore, when the cooling speed was 1.4 times the cooling oil, the crankshaft straightness was at a smaller level. It was found that the straightness of the crankshaft decreased significantly when the cooling speed increased. Both an increase in the tempering temperature and holding time had a decreasing effect on the straightness of the crankshaft, and the tempering temperature had a smaller impact on the straightness of the crankshaft than the holding time. 

**Figure 9:**
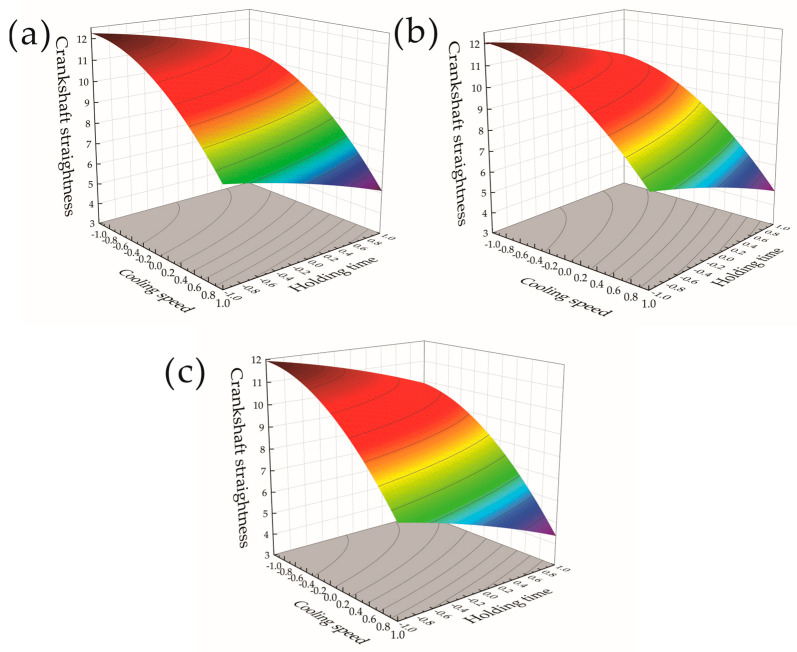
Response surface and contour plot of cooling speed and holding time to crankshaft straightness. (**a**) Tempering temperature of 500 °C. (**b**) Tempering temperature of 550 °C. (**c**) Tempering temperature of 600 °C.

Response surfaces and contour plots of the effect of the cooling speed and holding time on the crankshaft straightness at tempering temperatures of 500 °C, 550 °C, and 600 °C are shown in Figure 9a–c, respectively. When the tempering temperature gradually increased from 500 °C to 600 °C, the crankshaft straightness decreased as the temperature increased, indicating that the impact of the tempering temperature on the crankshaft straightness was negatively correlated. From the comparison of the three groups of response surfaces in Figure 9a–c, it was found that the cooling speed and holding time increased in the corresponding horizontal interval from −1 to 1 when the tempering temperature increased, which had an impact on the crankshaft straightness. The impact of the holding time and cooling speed on the crankshaft straightness was greater than that under low-temperature conditions. It can be seen from the analysis results that, when the tempering temperature was too low, the straightness of the crankshaft was too high. An increase in the tempering temperature reduced the straightness of the crankshaft, but when the tempering temperature was too high, it resulted in a weakening of the effect of the cooling speed and holding time on the straightness of the crankshaft, and therefore, an optimum range of tempering temperatures that allow for the best straightness to be obtained for the crankshaft exists. Controlling the tempering temperature within an optimal range can reduce the impact of the holding time and cooling speed on the crankshaft straightness and obtain the optimal crankshaft straightness results, which can provide certain theoretical guidance for actual production and processing.

**Figure 10:**
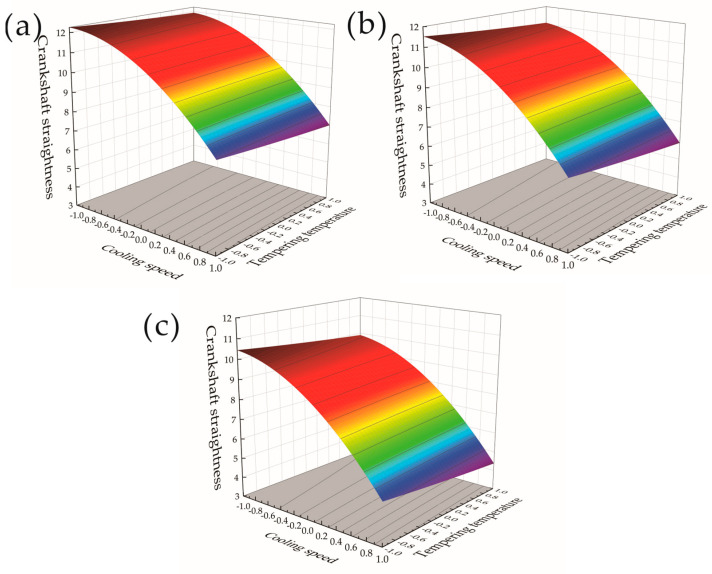
Response surfaces and contour plots of cooling speed and tempering temperature to crankshaft straightness. (**a**) Holding time of 4 h. (**b**) Holding time of 5 h. (**c**) Holding time of 6 h.

Figure 10a–c represent the response surfaces and contour plots of the effects of the cooling speed and tempering temperature on the crankshaft straightness when the holding time was 4 h, 5 h, and 6 h, respectively. It is found from the response surface in Figure 10a–c that the height of the response surface gradually became lower, indicating that the straightness of the crankshaft decreased with an increase in the holding time. From the three sets of response surface diagrams in Figure 10a–c, it was found that, along the tempering temperature direction, the crankshaft straightness gradually decreased when the tempering temperature level changed from −1 to 1. This decrease was within 0.5 mm, indicating that the tempering temperature was negatively correlated with the crankshaft straightness, but the degree of influence was small. As the tempering temperature and cooling speed increased, the crankshaft straightness continued to decrease and the cooling speed had a greater impact on the crankshaft straightness than the tempering temperature.

It can be seen from the variance analysis table and contour plot that, within a certain heat treatment range, the influence of each heat treatment parameter on the straightness of the crankshaft, from large to small, was: X1 cooling speed > X3 holding time > X2 tempering temperature. The degree of influence of the parameter interaction on the crankshaft straightness, from large to small, was: X1 cooling speed–X3 holding time > X1 cooling speed–X2 tempering temperature > X2 tempering temperature–X3 holding time. Both multi-factor coupling analysis and single-factor analysis showed that the cooling speed had the greatest impact on the heat treatment process. The consistent results of the two analysis indicate that the analysis results were relatively stable. In the process of crankshaft heat treatment, in order to obtain higher-quality parts, priority should be given to using a greater cooling speed and selecting an appropriate tempering temperature and holding time.

### 3.3. Optimization and Verification of Crankshaft Heat Treatment Process

Based on the above simulation experiment results and the experimental data of the response surface model, the parameters in each response surface were further optimized, that is, the optimal combination of process parameters was found under the condition that the crankshaft met the crankshaft straightness, surface stress, and martensite content. The response surfaces were analyzed using the Design-Expert software and the targets were set as the straightness of the crankshaft after heat treatment D < 3 mm, surface stress K > 400 MPa, and martensite content M > 0.8. The optimal process parameters selected were a cooling speed of 1.4 times the cooling, an oil and tempering temperature of 555 °C, and a holding time of 6 h. The best simulation results obtained were a crankshaft straightness of 2.59 mm, a surface stress of 526 MPa, and a martensite content of 0.8923.

**Figure 11:**
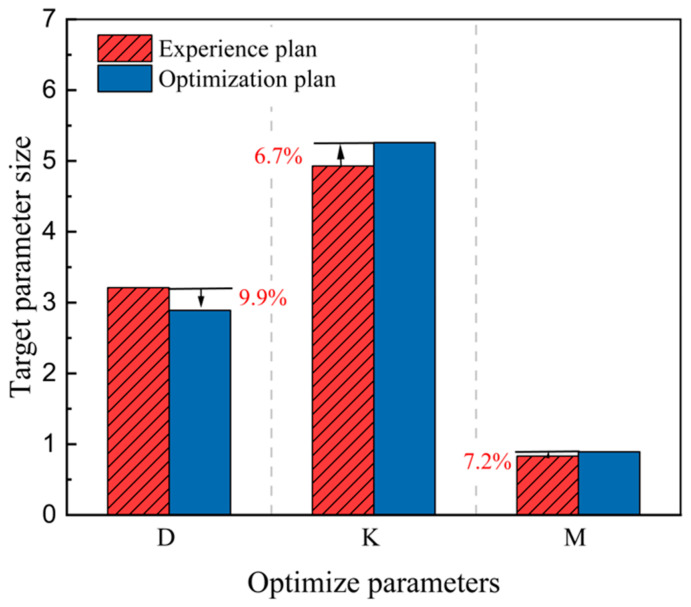
Comparison of solution optimization results.

A comparison between the empirical scheme and the optimized scheme is shown in Figure 11. The crankshaft straightness before optimization was 3.21 mm, and the crankshaft straightness after optimization was 2.89 mm. Compared with the empirical scheme, the crankshaft straightness was reduced by 9.9%. Before the optimization, the crankshaft straightness was 9.9%. The surface stress of the crankshaft was 493 MPa, and the surface stress of the optimized crankshaft was 526 MPa. Compared with the empirical scheme, the surface stress of the crankshaft increased by 6.7%. The martensite content before optimization was 0.8320, and the optimized scheme increased to 0.8923, while the martensite content increased by 7.2%. Figure 11 demonstrates a comparison of the changes in the optimized parameters corresponding to the empirical and optimized plan before and after the optimization of the process parameters. The crankshaft heat treatment process increased the surface stress and optimized the hardness on this basis, reducing the straightness of the crankshaft and making the overall performance of the crankshaft better. Compared with the empirical scheme, the optimized scheme gives certain guidance for the quenching and tempering heat treatment of crankshafts.

## 4. Conclusions

(1) The maximum value of the residual surface stress was at the outer surface of the crankshaft, with a maximum value of 462 MPa, the minimum value was at the front and rear end shafts of the crankshaft, with the minimum value tending to 0, and the overall deformation of the crank was small. The martensite mainly distributes on the surface of the crank with a maximum martensite content of 0.7639, and a lower content of martensite exists on the surface of the rod journal. The content of martensite was uniform in the region of rounded corners between the crank and the connecting rod journal.
(2) The residual strain, residual stress, and martensite content of the crankshaft after quenching and high-temperature tempering increased with an increase in cooling speed, tempering temperature, and holding time. The most prominent process factor affecting the mechanical properties, deformation, and structure during heat treatment was the cooling speed.
(3) The effects of the cooling speed, tempering temperature, and holding time process parameters on the crankshaft straightness, surface stress, and martensite content were studied. It was found that, under the influence of a single factor, the impact on each response value, from large to small, was: cooling speed > holding time > tempering temperature. Under the influence of multi-factor coupling of each parameter, the impact on each response value, from large to small, was: cooling speed–holding time > cooling speed–tempering temperature > holding time–tempering temperature.
(4) Through the structural optimization of the response surface method, the process plan for the quenching and tempering heat treatment was determined as follows: the cooling speed was 1.4 times the cooling oil, the tempering temperature was 555 °C, and the holding time was 6 h. According to the best results after the optimization, the crankshaft straightness was 2.89 mm, which was 9.9% lower than that before the optimization. The crankshaft surface stress was 526 MPa, which was 6.7% higher than that before the optimization. The martensite content of the crankshaft was 0.8923, and compared with that before the optimization, it increased by 7.2%.

## Figures and Tables

**Table 1:** Factor level table.

**F** **actor**	**Process Parameters**		**Level**	
		**−1**	**0**	**1**
X1	Cooling speed °C/s	1	1.2	1.4
X2	Tempering temperature °C	500	550	600
X3	Holding time h	4	5	6

**Table 2:** Experimental plan and simulation results.

Factor Level Coding				Evaluation Index		
Serial Number	X1	X2	X3	Straightness	Residual Stress	Martensite Content
				mm	MPa	%
1	−1	0	1	10.11	419	0.6219
2	−1	0	−1	12.5	411	0.5936
3	−1	1	0	10.96	406	0.5861
4	0	1	−1	10.28	426	0.6642
5	0	0	0	9.83	462	0.7623
6	0	−1	−1	10.84	421	0.6378
7	1	1	0	6.15	486	0.8321
8	1	0	1	4.27	512	0.8795
9	0	−1	1	9.21	439	0.6923
10	0	0	0	9.83	462	0.7623
11	0	0	0	9.83	462	0.7623
12	0	0	0	9.83	462	0.7623
13	0	1	1	8.42	454	0.7235
14	1	0	−1	7.26	493	0.8031
15	0	0	0	9.83	462	0.7623
16	1	−1	0	6.63	482	0.8264
17	−1	−1	0	11.25	401	0.5752

**Table 3:** Comparison of regression model fitting accuracy.

		Linear	2FI	Quadratic
*D*	R^2^	0.9072	0.9088	0.9881
	R^2^_adj_	0.8858	0.8541	0.9727
	R^2^_pre_	0.9302	0.6331	0.8090
*K*	R^2^	0.8704	0.8736	0.9950
	R^2^_adj_	0.8404	0.7978	0.9887
	R^2^_pre_	0.7654	0.5033	0.9207
*M*	R^2^	0.8758	0.8800	0.9976
	R^2^_adj_	0.8471	0.8080	0.9946
	R^2^_pre_	0.8002	0.6338	0.9619

**Table 4:** Model significance judgment.

*p* Value	Explain
*p* < 0.01	Significantly
0.01 < *p* < 0.05	More significant
0.05 < *p* < 0.1	Moderate
*p* > 0.1	Not significant

**Table 5:** Variance analysis of crankshaft straightness.

Source	Sum of Squares	df	Mean Square	*F*	*p*
Model	68.59	9	7.62	64.36	<0.0001
A	52.58	1	52.58	444.07	<0.0001
B	0.5618	1	0.5618	4.74	0.0658
C	9.83	1	9.83	83.06	<0.0001
AB	0.0090	1	0.0090	0.0762	0.7905
AC	0.0900	1	0.0900	0.7601	0.4122
BC	0.0132	1	0.0132	0.1117	0.7480
A^2^	5.26	1	5.26	44.41	0.0003
B^2^	0.0052	1	0.0052	0.0436	0.8406
C^2^	0.1327	1	0.1327	1.12	0.3250
Residual	0.8289	7	0.1184		
Lack of Fit	0.8289	3	0.2763		
Pure Error	0.0000	4	0.000		
Cor Total	69.42	16			

**Table 6:** Surface stress variance analysis.

Source	Sum of Squares	df	Mean Square	*F*	*p*
Model	1.70	9	0.1891	156.16	<0.0001
A	1.41	1	1.41	1165.59	<0.0001
B	0.0105	1	0.0105	8.68	0.0215
C	0.0666	1	0.0666	55.02	0.0001
AB	0.0000	1	0.0000	0.0206	0.8898
AC	0.0030	1	0.0030	2.50	0.1580
BC	0.0025	1	0.0025	2.06	0.1939
A^2^	0.0032	1	0.0032	2.63	0.1489
B^2^	0.1857	1	0.1857	153.37	<0.0001
C^2^	0.0152	1	0.0152	12.52	0.0095
Residual	0.0085	7	0.0012		
Lack of Fit	0.0085	3	0.0028		
Pure Error	0.0000	4	0.000		
Cor Total	1.71	16			

**Table 7:** Variance analysis of martensite content.

Source	Sum of Squares	df	Mean Square	*F*	*p*
Model	1399.90	9	155.54	326.26	<0.0001
A	1162.34	1	1162.34	2438.04	<0.0001
B	6.88	1	6.88	14.44	0.0067
C	59.68	1	59.68	125.18	<0.0001
AB	0.0676	1	0.0676	0.1418	0.7177
AC	5.78	1	5.78	12.13	0.0102
BC	0.0576	1	0.0576	0.1208	0.7384
A^2^	1.59	1	1.59	3.33	0.1109
B^2^	110.43	1	110.43	231.63	<0.0001
C^2^	42.14	1	42.14	88.40	<0.0001
Residual	3.34	7	0.4768		
Lack of Fit	3.34	3	1.11		
Pure Error	0.0000	4	0.0000		
Cor Total	1403.23	16			

## Data Availability

The raw data supporting the conclusions of this article will be made available by the authors on request.
